# High Spatial Resolution Building Characteristics for the Global South: Insights from the Google Open Buildings Temporal Dataset (2016-2023)

**DOI:** 10.12688/gatesopenres.16386.1

**Published:** 2026-06-02

**Authors:** Rhorom Priyatikanto, Heather Chamberlain, Maksym Bondarenko, Wenbin Zhang, Natalia Tejedor Garavito, Andrew Tatem

**Affiliations:** 1 University of Southampton School of Geography and Environmental Science, Southampton, England, SO17 1BJ, UK

**Keywords:** built environment, building characteristics, geospatial data

## Abstract

**Background:**

The need for detailed built-up area data for applications such as population modelling, urban planning, and environmental research is growing due to the pace of global population changes, particularly in the Global South, where existing datasets have limitations.

**Methods:**

Here, we processed the Google Open Buildings Temporal (OBT) dataset to derive six 100-m spatial resolution datasets per year on building characteristics. The characteristics include building count, total perimeter, total area, total volume, height variance, and mean distance to the nearest building edges. These were calculated using arithmetic operations, convolutions, and spatial aggregation. The derived data was validated against a set of existing largescale open spatial datasets on buildings and human settlement extents for single timepoints. Additionally, temporal consistency was assessed, with polynomial fitting explored to test suitability for smoothing the data where significant fluctuations were seen.

**Results:**

The new dataset strongly correlated with the Google Open Buildings Polygons dataset (e.g., building count: *r* = 0.88; building area: *r* = 0.90) but showed systematic perimeter underestimation in dense areas due to blending effects. Weaker correlations were found with other datasets due to methodological differences. Internally, building height variance correlated moderately with total volume ( *r* = 0.47). A strong positive correlation ( *r* > 0.8) existed between building count, area, volume, and population. Temporal analysis revealed significant fluctuations in most characteristics, especially height-related metrics, with second-order polynomial fitting proving optimal for smoothing.

**Conclusions:**

A validated 100-m resolution building characteristics dataset for the Global South, covering each year from 2016 to 2023, derived from Google OBT, was produced. While showing consistency with similar largescale spatial datasets, temporal fluctuations indicate a need for further processing for time-series applications.

## Background & Summary

The global population’s surge past 8 billion in 2022, with projections reaching 10 billion by 2080, profoundly impacts the natural and built environments. ^1^^,^^2^ This rapid demographic shift intensifies the demand for comprehensive data on built-up areas, infrastructure, and land use across all geographic scales. Such data is critical for strategic decision-making that shapes our world.

Information on the distribution of built-up areas guides resource allocation, informs investment decisions in infrastructure, and helps track economic growth and development across regions. The patterns of the built environment are also crucial for assessing vulnerability to natural disasters like floods, ^3– 5^ earthquakes, ^6^ and volcanoes. ^7^ Data on infrastructure and building density informs emergency preparedness plans, evacuation procedures, and resource deployment during disasters. ^8^ Knowing the size, characteristics, and distribution of built-up areas allows for targeted interventions to address issues like sustainable urbanization, ^9^ access to essential services, ^10^ and responsible consumption. ^11^^,^^12^

Population and demographic changes exert significant pressure on built environments, driving the demand for housing, infrastructure, and essential services. This demand fuels urban expansion, redevelopment initiatives, and the intensification of land use. Conversely, in some contexts, the expansion of built-up areas is outpacing population growth. ^13^ Increased population density often leads to vertical growth as cities build upwards to accommodate more people. In contrast, declining populations and persistent economic recession can lead to urban shrinkage, characterised by vacant spaces and underutilised infrastructure. ^14^^,^^15^ This underscores the critical need for comprehensive and accurate global human settlement data at high resolutions to effectively monitor, plan, and manage the complex interplay between population dynamics and the urban landscape.

Several global datasets representing built settlement have been produced from multispectral and multitemporal remote sensing data, especially those from the Landsat and Sentinel satellites. Example datasets include the Global Urban Footprint (GUF), ^16^^,^^17^ the Global Human Settlement Layer (GHSL), ^18^^,^^19^ the World Settlement Footprint (WSF), ^20^ and the Global Impervious Surface Area (GISA). ^21^ The spatial resolution of these gridded datasets varies from fine to moderate resolution (10 to 500 metres), with grid cell values representing the presence/absence of buildings, or summary metrics such as built settlement density or area. Other datasets representing a broad range of land cover classes, also include built settlement. ^22^ Recent advancements, utilizing elevation data from sources such as the Shuttle Radar Topography Mission, Advanced Land Observing Satellite, and TanDEM-X, have enabled datasets like GHSL and WSF-3D ^23^ to incorporate 3D attributes such as building height and volume.

In addition to gridded settlement data, the last five years has seen rapid growth in the availability of multi-country building footprint datasets, providing vector polygon outlines of individual buildings, at scale. ^24^ These datasets, extracted from satellite imagery, ^25^ provide detailed data on the location, shape and size of individual buildings, enabling greater insights into spatial patterns of buildings and urban morphology. Of the openly-published building footprint datasets, most are produced through automated feature extraction from high-resolution satellite imagery. Examples include Global Building Atlas, ^26^ Microsoft Building Footprints, Google Open Buildings, ^27^ and EUBUCCO. ^28^ Alternatively, building footprints may be manually digitised based on visual interpretation of satellite imagery, such as is done by the OpenStreetMap community. As vector polygon data, building footprint datasets provide outlines of buildings that can be used at a range of geographic scales. When working with building footprint data across large geographic extents, or when there is a need to integrate with other gridded datasets (e.g. flood inundation extents), data can be summarised by calculating building metrics in gridded format. ^29^^,^^30^ For example, simple metrics on building count, area, and perimeter, as well as metrics relating to distance between buildings, compactness and shape, ^31^ which are relevant in many contexts include population density estimation ^32^^,^^33^ and urban planning. ^34^

Nevertheless, the current landscape of global building footprint data is defined by a stark digital divide, where data availability remains critically low in the regions that need it most. Research consistently shows that OpenStreetMap (OSM) completeness falls below 20% for thousands of cities encompassing nearly half the global urban population. Most of the severe gaps are concentrated in the Global South. ^35, 36^ This scarcity is often rooted in structural barriers, such as the high cost of commercial satellite imagery and the absence of robust civil registration systems. ^37, 38^ Even when multiple open-access datasets are available, they often display massive inconsistencies in building counts and area coverage, complicating their fitness-for-purpose for urban planning and population modeling. ^39^

These availability gaps create significant spatial and socio-economic biases, as datasets often favor high-income countries while omitting informal settlements and remote rural areas. ^37, 40^ When these flawed snapshots are used to train artificial intelligence or guide humanitarian efforts, the bias is amplified. DNN models show significantly lower accuracy in impoverished areas, and health interventions risk excluding vulnerable populations. ^41^ Such systemic omissions highlight that static, incomplete datasets fail to capture the rapid, fluid changes inherent to urban growth in the Global South.

On the other hand, settlement data with sufficient spatial and temporal resolution is needed for many applications. However, most high-resolution settlement datasets are limited in their temporal coverage. GHSL provides data at five-year intervals from 1975 to 2030, while WSF exhibits temporal latency, with its most recent epoch in 2019. The Google Open Buildings Temporal (OBT) dataset aims to address these limitations. ^42^ The dataset consists of very high-resolution annual raster on building presence, fractional count and height, covering the Global South, with data annually for 2016 to 2023. Leveraging Sentinel-2 imagery, OBT offers 3D building information at a nominal 50-cm resolution, with an effective resolution of 4 m. This dataset is a step change, being the first temporally explicit built settlement dataset, with multiple-continent coverage, providing rich information practically at building-level. From the three layers in the dataset (building presence, fractional count, and height), a range of metrics characterising the built environment can be derived, enabling these to be mapped across continents for multiple annual timepoints for the first time.

The very high spatial resolution of the Google OBT dataset is advantageous in providing detailed data at close to the level of individual buildings, however it also provides computational challenges for working with the data across large spatial extents. In the meantime, raster data with slightly lower-resolution is essential for multifaceted studies ranging from regional to global in scope. For example, the Worldpop Global Demographic Data Project requires settlement data as an ancillary variable for global population distribution modelling. Temporal data is also needed to understand the dynamic sprawl of settlement area, which is in line with population growth. ^43, 44^ Without discounting other potential applications, ^45– 47^ the need for data for population distribution modeling is the primary motivation behind our current work.

In this paper we describe a set of data on building characteristics at 100-m resolution derived from the Google OBT dataset, from 2016 to 2023. In total, we produced dataset containing 48 layers describing annual building count, total area, volume, perimeter, height variance, and mean distance for the Global South. The first three parameters are the products of simple aggregation of the input data and are commonly available in existing datasets (GHSL, WSF-3D, etc.) so that comparison between our data product and those datasets can be done to ensure its validity. Building perimeter is valuable in the study of building energy performance and urban climate ^48^^,^^49^ so that derivation of this parameter will be useful for urban planning and environmental studies. It is also a valuable urban morphology indicator that influences diffusion of anthropocentric heat in the city. ^50^ Additionally, variation in building heights also captures spatial inequalities. ^51^ Lastly, the mapping of mean distance to building is a contextual metric that also measures built-up density and its possible future expansion.

## Materials and methods

This section provides an overview of the Google OBT dataset, which serves as the input for generating gridded building characteristics layers at 100-m resolution. The processes involved—including arithmetic calculations, convolutions, and spatial aggregation—vary depending on the specific layer being produced. Additional processing steps, such as mosaicking and clipping by country boundaries, are also described.

### Overview on google open buildings temporal

**Figure 1.**
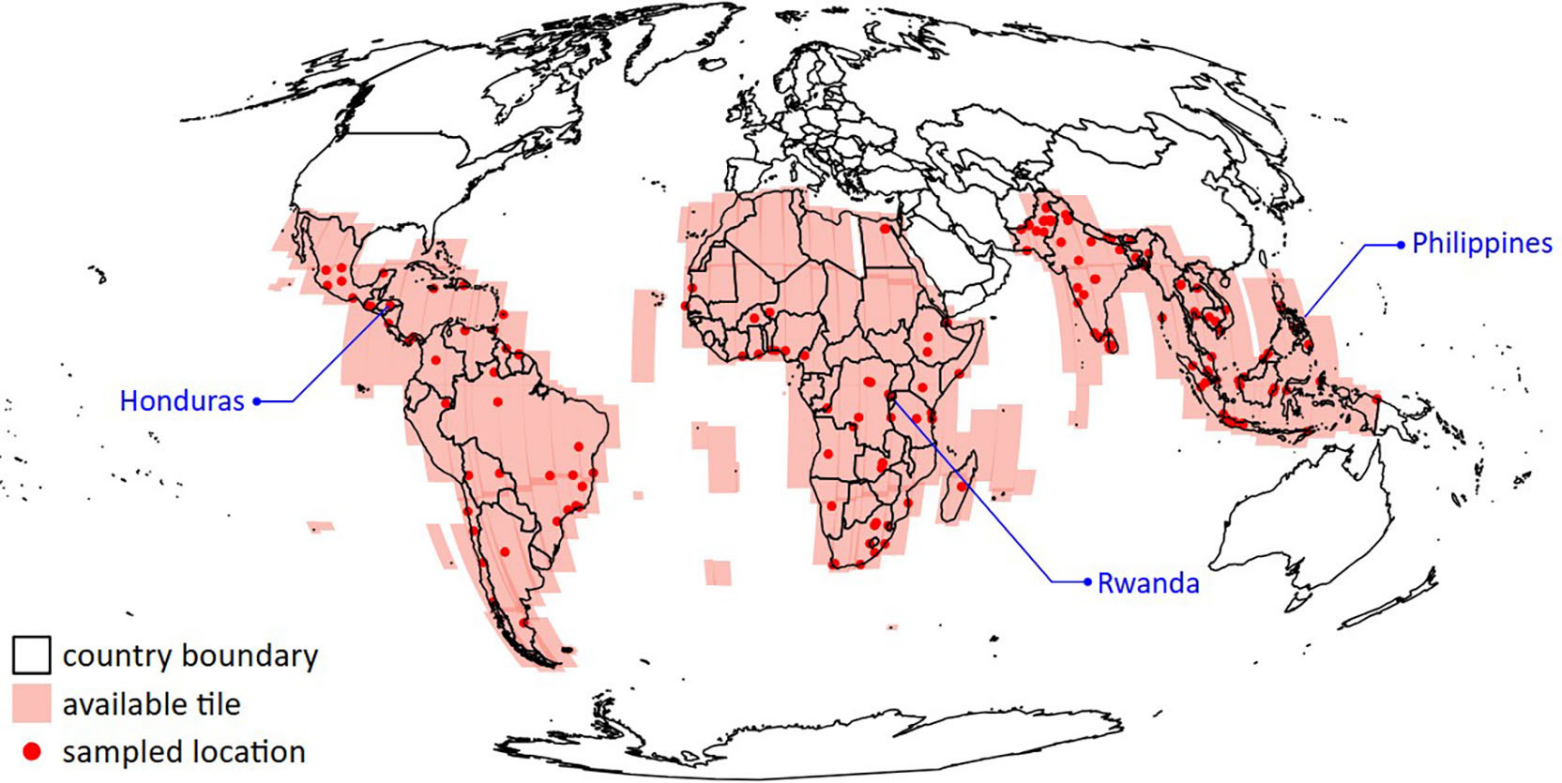
The coverage of google OBT on the global south (pink tiles). The building statistics for three countries (annotated) were validated by comparing them against population data at the subnational level. Locations selected for technical validation are marked as red dots.

Google Open Buildings Temporal Dataset ^42^ is a collection of data showing how building presence, counts, and heights have changed over time in many parts of the world. It provides annually snapshots from 2016 to 2023 at a spatial resolution of 50 cm. Focusing on the Global South, the dataset covers areas in Africa, South and Southeast Asia, Latin America, and the Caribbean (see Figure 1).

To identify buildings, the Google Research team employed a deep learning technique ^42^ to analyse a large corpus of Sentinel-2 imagery. A key task of this approach was building segmentation, generating both confidence masks and building centroid locations. To overcome the limited availability of human-derived building labels, a teacher-student learning framework ^52^ was employed. ^27^ The teacher model, which was a large and computationally exhaustive model, trained using high-resolution (50 cm) imagery and existing human labels, generated additional training data for the student model. This effectively expanded the training dataset. The student model then performed building super-resolution segmentation using stacks of 32 Sentinel-2 images (10-m resolution) acquired around June 30 each year (typically 16 images before and 16 after). Multi-temporal stacks were proven to enhance segmentation accuracy, e.g., increase of mean intersection over union, from 72% to 77%, when full-stacks were used in the training instead of single-timeframe images. ^42^ However, cloud cover frequently resulted in incomplete image stacks, potentially degrading the model’s performance.

Building height prediction followed a similar teacher-student approach. Height labels were derived by calculating the difference between the Digital Surface Model and the Digital Terrain Model, effectively isolating above-ground object height. ^53^ This approach captured the heights of both natural and man-made features. Therefore, to isolate building heights, a corresponding building confidence layer, derived from the segmentation process, was used to mask out non-building objects. Validation against ground truth data from North America, Europe, and Japan yielded a mean absolute error of 1.5 m for building height predictions. ^42^ This indicates a reasonable level of accuracy across diverse geographic contexts, although further regional validation may be warranted.

The overall process yielded three output layers, accessible via Google Earth Engine ^54^: building presence, fractional count, and building height, all at a native resolution of 50 cm. The building presence layer, with values ranging from 0 to 1, represents the model’s confidence that a given grid cell belongs to a building. The fractional count layer (values ranging from 0 to approximately 0.2) encodes building centroid information; the grid cell with the highest fractional count within a building’s footprint corresponds to its centroid. Integrating the fractional count over a given area provides an estimate of the number of buildings within that area. Finally, the building height layer represents the height of buildings above ground level, capped at 100 m.

### Computed layers

We computed six 100-m resolution layers characterising buildings for each year covered by the OBT dataset: building count, total perimeter, total footprint area, total volume, variance of building heights, and mean distance to buildings. For each layer, we computed annual data from 2016 to 2023. From these characteristics, other indicators can be derived, e.g., mean building heights, mean number of storeys, and the average complexity index. ^31^

**Figure 2.**
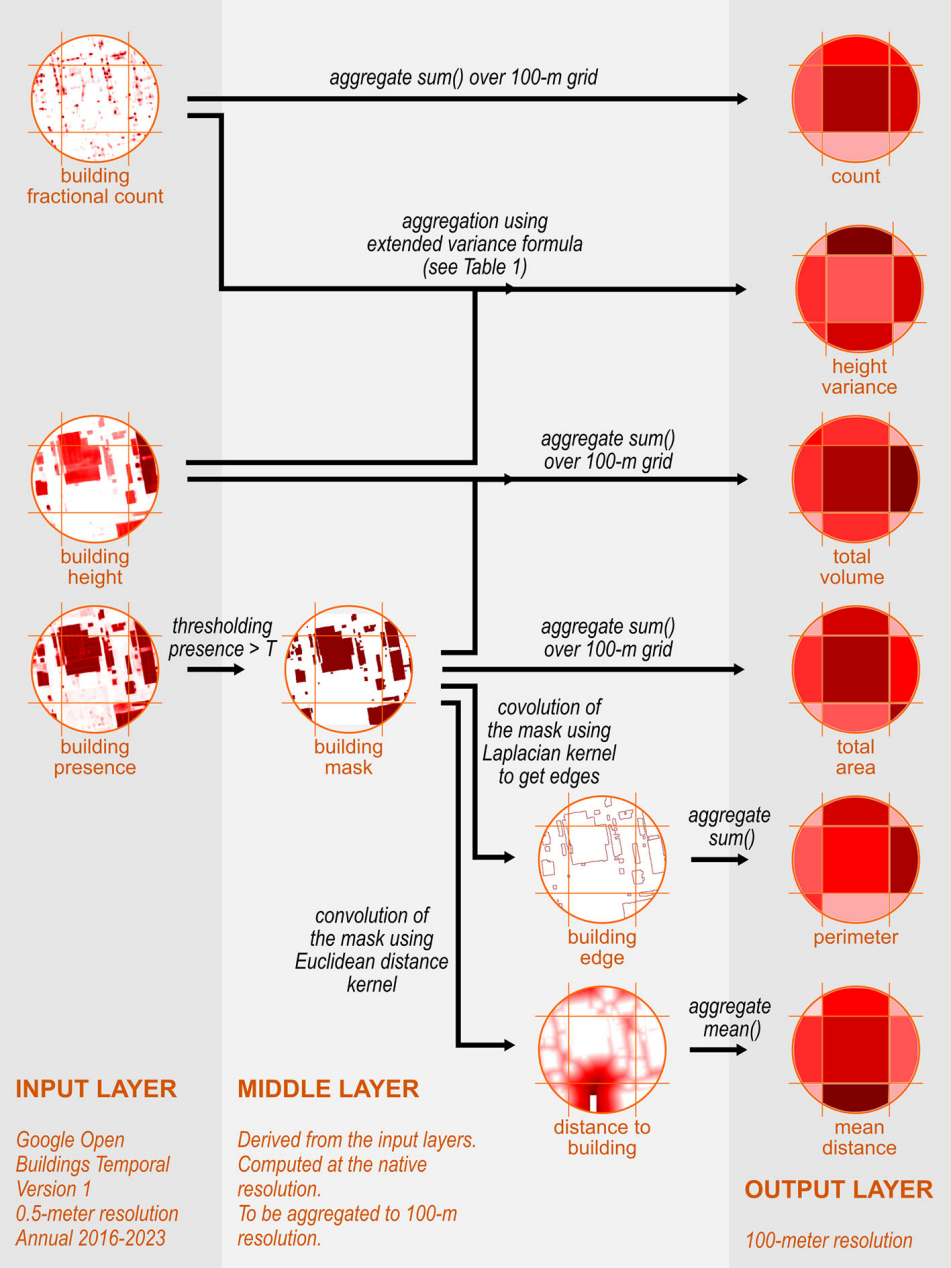
Workflow from OBT layers to building characteristics at 100-m spatial resolution.

As illustrated in Figure 2, we utilised three layers from the OBT dataset and perform several processes, including thresholding, arithmetic calculation, convolution, and aggregation. Table 1 summarises input used and output layers produced in this study. To focus on building characteristics, we applied a masking procedure, excluding grid cells with building presence values below a threshold *T.* We explored three thresholds: *T* = 0.3, 0.4, and 0.5, resulting in three sets of output layers. While the Google Research team suggests a threshold of *T* = 0.34, acknowledging that the optimal value may vary by region, our approach allows for a broader analysis. This initial masking step was essential to focus specifically on building characteristics.

**Table 1.** Description of the output layers and the formulae to produce them. *R _s_* () and *R _m_* () respectively are functions to reduce resolution using sum and mean reducers.

Layer	Unit	Range	Formula
Count (n)	-	0 – 1000	$n=40,000\times R_{s}\left(\text{frac}\right)$
Total perimeter (p)	m	0 – 1000	$p=2,000\times R_{s}\left(\text{edge}\right)$
Total area (A)	m ^2^	0 – 10000	$A=10,000\times R_{s}\left(\text{pres}>T\right)$
Total volume (V)	10 m ^3^	0 – 65535	$V=1,000\times R_{s}\left(\text{height}\right)$
Variance of height (varh)	m ^2^	0 – 65535	$\text{varh}=\frac{R_{m}\left({\text{frac}}_{i}{h_{i}}^{2}\right)}{R_{m}\left({\text{frac}}_{i}\right)}-{\left(\frac{R_{m}\left({\text{frac}}_{i}h_{i}\right)}{R_{m}\left({\text{frac}}_{i}\right)}\right)}^{2}$
Mean distance (d)	0.01 m	0 – 566000	$d=100\times R_{m}\left(\left(\text{pres}>T\right)*\text{kernel}\right)$

Building count was simply the integral of building fractional counts ( *frac*) in 100-m grid cells. A multiplication factor of 40,000 was used to adjust the total count obtained using reduceResolution() function in GEE. This factor was associated with the pyramiding policy implemented, i.e., the multiplication factor was the number of original 50-cm grid cells inside the final 100-m cell. Almost similar to this process, the sum of grid cells with building presence ( *pres*) above *T* multiplied by 10,000 yielded the total area covered by the building in m ^2^. Next, replacing the confidence mask with the masked building height layer ( *h*) prior to the aggregation yielded total building volume in m ^3^. For practical reasons, the total building volume layer was multiplied by 0.1. All above layers were stored as 16-bit unsigned integers rasters, which provide the optimal balance between data size and dynamic range for the extracted values.

Calculating the total building perimeter required a different approach. Typical building footprint extraction involves segmenting the image, then vectorizing the segmented images and applying morphological corrections to fit simplified polygons, resulting in vector-based building footprint data. ^55^ However, because we were aggregating building characteristics to a lower resolution, we skipped the vectorization step and directly calculated the perimeter from the edges from the building confidence mask. We used a Laplacian-8 kernel to perform edge detection on this mask. Among several kernels available for edge detection in Earth Engine (e.g., Prewitt, Roberts, Sobel), Laplacian-8 was selected due to its sensitivity to the changes in image intensity and its ability to detect edges in all directions equally. ^56^ Lastly, the sum of the detected edge grid cells then provided our total building perimeter value for each 100-m cell.

Variance of building heights in each grid cell was computed using the formula summarised in Table 1. While a direct calculation of variance from individual building heights was possible, it was computationally burdensome. To reduce this computational effort, we adopted an alternative calculation: variance equals to the average of the squared height minus the square of the average height. Critically, the averaging part utilised the building fractional count as a weighting factor. This technique correctly isolated the contribution of each building’s height, preventing the building’s spatial area from unduly influencing the variance calculation.

Building proximity was calculated in Google Earth Engine by applying a Euclidean distance transform with a 400-m kernel radius to a defined building confidence mask. This process generated a raster representing the distance from each grid cell to the nearest building edge. Applying a radius limit of 400-m enabled efficient computation without losing too much information on sparsely built areas. Subsequently, the resulting distance raster was aggregated from 50-cm original resolution to a 100-m resolution grid using a mean reducer, providing the mean distance to buildings for each grid cell.

### Additional processing

Six layers were computed tile-by-tile using Earth Engine Python API. This approach facilitated processing large datasets. The resulting EPSG:4326 raster were downloaded for local processing, including creating global mosaic and clipping to country boundaries that conformed to the WorldPop Global Demographic Data Project’s master grid ^57^ for consistency.

## Dataset validation

Validation stage was conducted by visual inspection and internal consistency checks, comparison with existing largescale datasets and also comparison with population estimates. Additional assessment on temporal fluctuation and the effect of using different thresholds were also performed.

**Figure 3.**
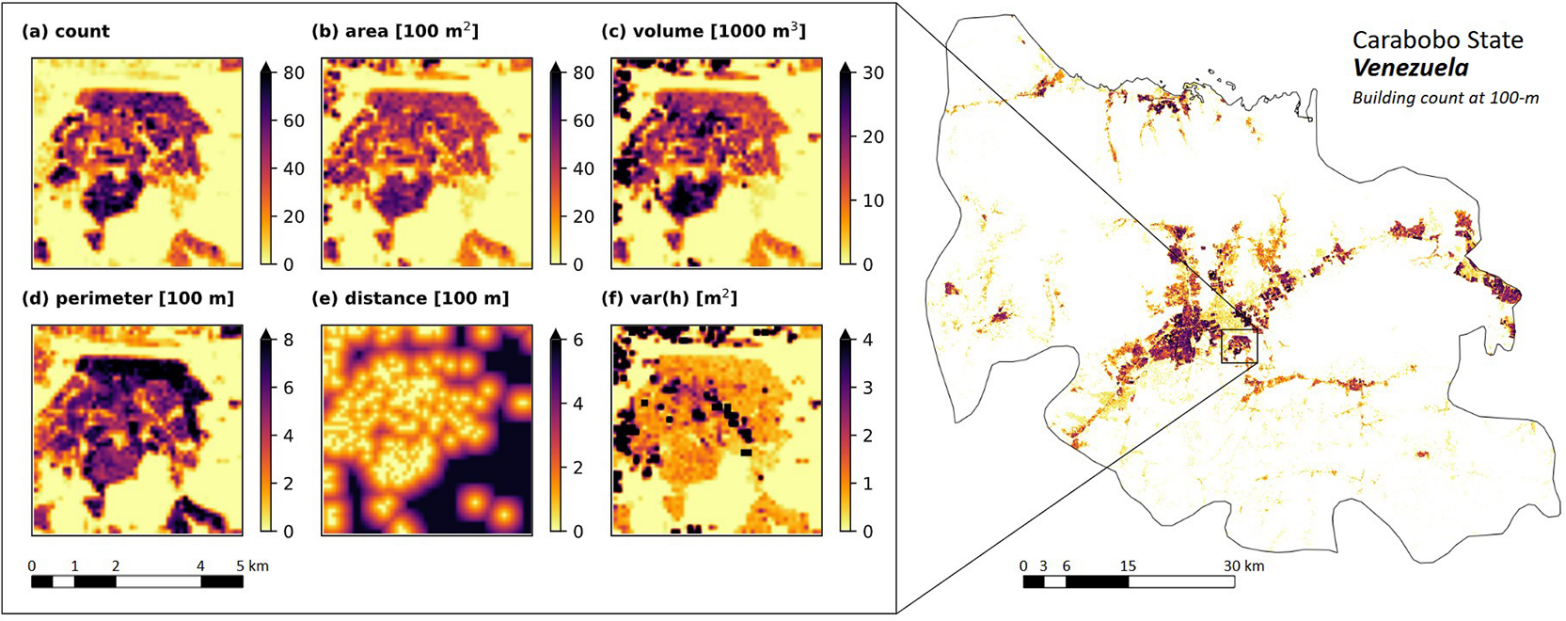
Building characteristics in a 5x5 km ^2^ area around Flor Amarillo, Valencia, Carabobo State, Venezuela.

Instead of inspecting all data, we extracted data associated with 5 × 5 km ^2^ area from 183 geographically-stratified sample locations (see Figure 1) with non-zero building counts and performed validation on this sample. The locations were selected to fairly represent diverse countries in the Global South and different degree of urbanisations. In practice, random locations were selected around major cities in the Global South and outside arbitrary urban area where buildings were more sparsely distributed. Utilising WorldPop Global Population Data for year 2020, our sampled locations represent diverse population densities, spanning from less than 100 to 30,000 individuals per square kilometre (Q _1_ = 1,100; Q _2_ = 3,000; Q _3_ = 7,200). An example of the selected area is displayed in Figure 3.

**Figure 4.**
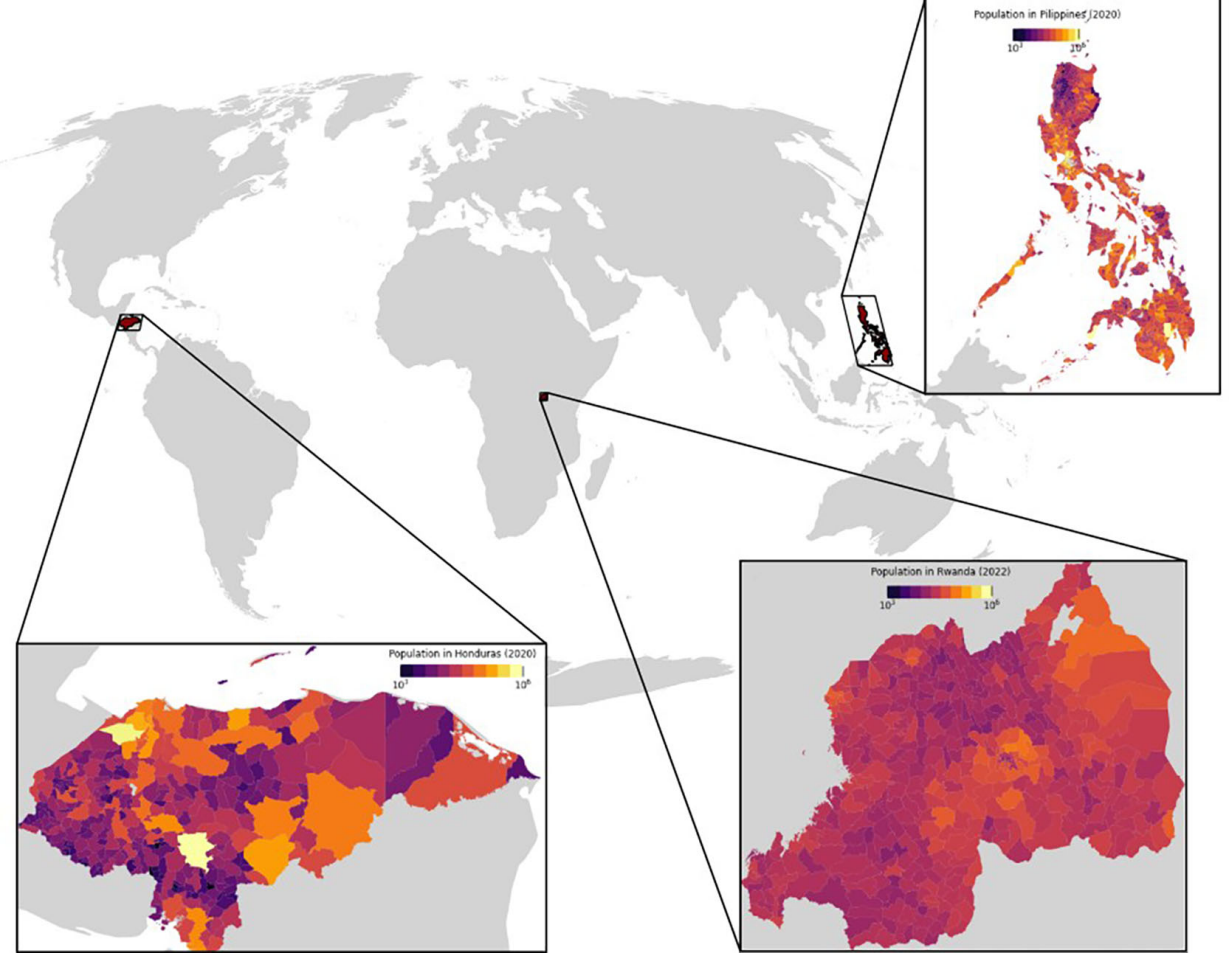
Population estimates at administrative level in three selected countries: Honduras, Rwanda, and the Philippines.

As another way to check the validity of the datasets, we evaluated the relationship between the building characteristics and population estimates in a selection of countries. This approach is grounded in the findings of Nieves et al., ^58^^,^^59^ who found that built settlement data was a strong predictor of population density. For this purpose, building count, area, and volume were aggregated at administrative unit level and compared with census data. Considering their socio-economic and demographic characteristics relative to the world average and the availability of recent census counts matched to subnational boundaries, we selected Honduras, Philippines, and Rwanda as the test countries. Recent census data on population counts and associated administrative boundaries of those countries were acquired from City Population (see the map in Figure 4 and the summary in Table 2). This source provides population statistics for countries, administrative divisions, cities, urban areas, and agglomerations around the world, obtained from official sources such as National Statistics Offices. The data for Philippines and Rwanda were based on the most recent censuses at the time of writing, while Honduras data was a 2020-projection based on the 2001 and 2013 censuses.

**Table 2.** Summary of the administrative unit-level population data to explore the relationships between aggregated building characteristics and population counts.

	Honduras	Philippines	Rwanda
Administrative level	Level-2 (municipalities)	Level-3 (municipalities)	Level-3 (sectors)
Number of units	298	1642	415
Median unit area	195 km ^2^	118 km ^2^	48 km ^2^
Census year	2020 (projection)	2020	2022
Total population	9.3 million	109.1 million	13.1 million

### General characteristics

Figure 3 depicts the spatial distribution of building characteristics derived from OBT for a 25 km ^2^ region encompassing Flor Amarillo, Carabobo State, Venezuela. The visualization highlights: (1) a densely settled area in the southern sector, characterised by a high concentration of buildings; (2) commercial zones in the northern and eastern sectors, distinguished by above-average total building volume; and (3) a central band displaying substantial height variance, indicative of apartment and commercial complexes surrounded by low buildings. This analysis provides insight into the diverse urban morphology of the region.

**Figure 5.**
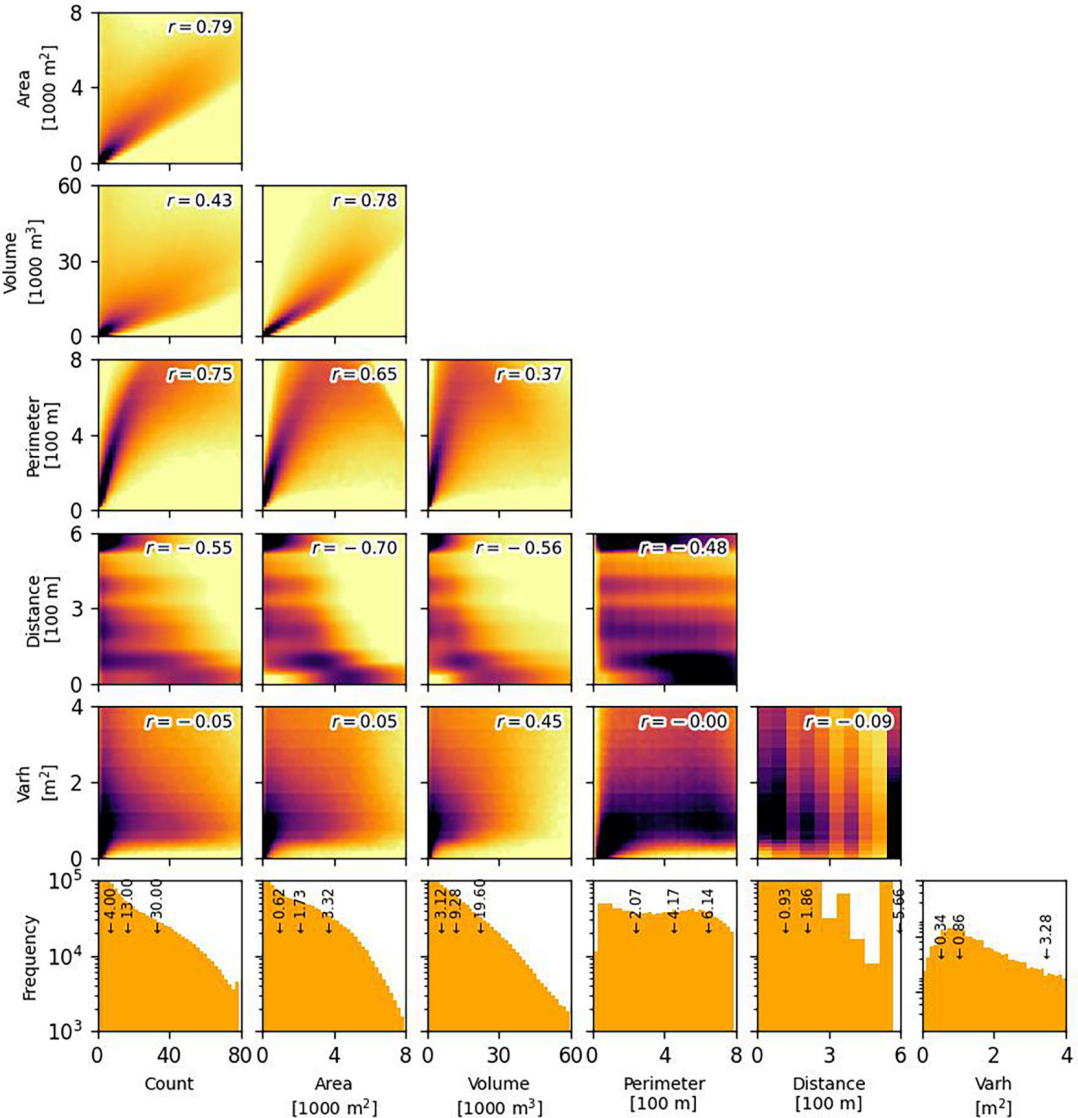
Density plots (darker means denser) highlighting correlations between building characteristics in 2020 (0.5 threshold) from 183 sample locations. Pearson’s r is indicated at the top-right corner of each panel. The bottom panels display a univariate distribution of the characteristics with Q _1_, Q _2_, and Q _3_ pinned on top of it.

To understand more, we checked the distribution of the building characteristics from the 183 sampled locations and assess the relationships between different characteristics. The characteristics were derived using threshold of 0.4. For every pair of building characteristics, we computed Pearson’s correlation coefficient ( *r*) as a statistical measure of correlation. Accordingly, the associated univariate and bivariate distributions are depicted in Figure 5.

In general, total area is the metric with the highest correlation with other characteristics (| *r*| > 0.6, except with height variance). This result is consistent with a simple expectation that the increase in built-up total area usually aligns with the increase of building count, perimeter, and volume. In more dense regions where built-up total area is larger, the average inter-building distance is lower.

Analysis reveals moderate to strong correlations among building count, total perimeter, total volume, and inter-building distance. The relationship between total area and perimeter exhibits a quadratic trend, with increase of data scatter at higher total areas. Meanwhile, lack of data points in the upper-right quadrant is observed, indicating potential limitations. Closely spaced buildings lead to perimeter underestimation due to blending effects during extraction. When adjacent structures are identified as overlapping, their shared boundaries are lost, reducing the calculated perimeter. While geometric edge detection, such as the Hough transform (San & Turker 2010), could alleviate these blending effects, we intentionally omitted this step to focus on broader 100-m grid aggregations and avoid computational burden. Furthermore, inter-building distance displays discrete values, a direct result of the 400-m radius distance kernel used in calculations. Spatial aggregation further reduces the granularity of these distance values.

Building height variance exhibits a distinct behaviour, showing minimal correlation with most other building characteristics. A moderate positive correlation exists between height variance and total volume ( *r* = 0.45), suggesting that areas with larger buildings tend to display greater height variation. Conversely, the correlation between height variance and mean building height (i.e., total volume divided by total area) is considerably weaker ( *r* = 0.28), indicating that average building height has less influence on height variability.

Univariate distributions reveal typical characteristics across sampled locations: a median building density of 14 buildings per hectare and a median building size of 150 m². The median building height, estimated at 5.1 m, approximates a two-storey structure. Assuming random sampling, these values offer a generalised representation of average building conditions within the Global South.

### Comparison with other datasets

This study assessed a dataset of 183 randomly selected locations across the Global South ( Figure 1). For each 5x5 km² area around the designated locations, we extracted building characteristics from our dataset and compared them with the following established datasets:

• Google Open Buildings Polygons v3 (Google Polygons): Building count, perimeter, and area were derived from the latest version of the Google Open Buildings Polygons, ^26^ a product of deep learning analysis of Sentinel-2 imagery (circa 2020). We used the rasterised and harmonised version of the building dataset prepared for the WorldPop Global Demographic Data Project. ^57^ Only buildings with a confidence score above 0.75 were used during rasterisation.
• Microsoft Building Footprint (Microsoft): Building count, perimeter, and area were derived from the Microsoft Building Footprint dataset, which contains 1.4 billion building footprints globally. This dataset was based on satellite imageries acquired between 2014 to 2021. While this dataset includes building height data, it is limited to North America, Europe, and Australia, and therefore not relevant to this Global South focused study. Rasterization process similar to that of Google Polygons was performed to Microsoft data. ^57^
• Global Human Settlement Layer (GHSL): Building area and volume were obtained from the 2020 GHSL dataset. ^19^ This raster dataset was reprojected to the coordinate reference system used by the WorldPop Global Demographic Data Project. ^57^ This harmonised dataset also incorporates non-residential building footprints from OSM.
• World Settlement Footprint 3D (WSF3D): Building count, area, and volume in 2019 were derived from the WSF3D dataset. ^23^ This 90-m resolution raster was resampled using the cubic method and reprojected to match our study’s coordinate reference system.

**Figure 6.**
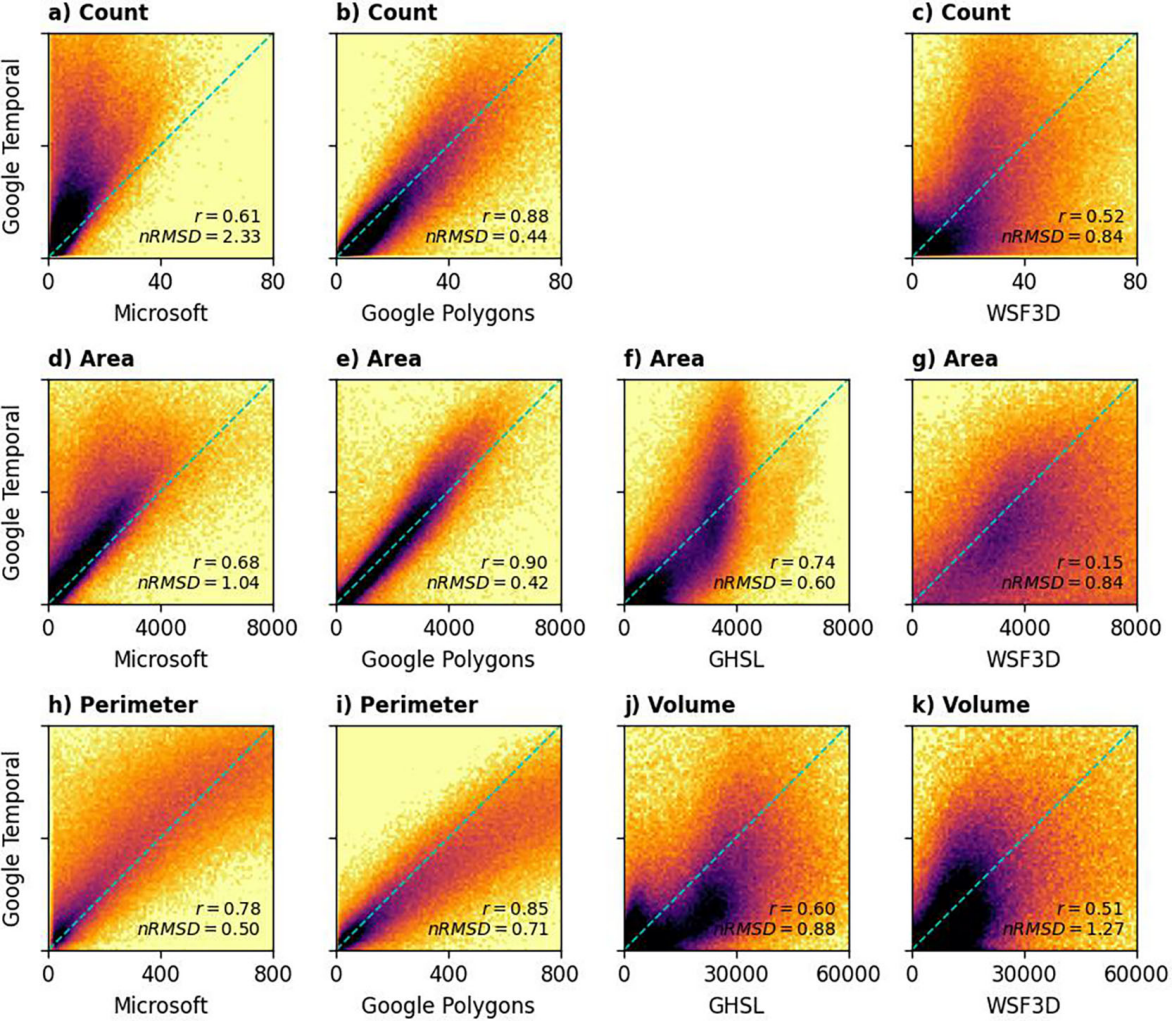
Comparison between building characteristics from 183 sample locations derived from OBT (vertical axis) and other datasets (horizontal axis). The density plots are scaled logarithmically to increase clarity.

The building characteristics for 2020 were compared against Google Polygons, Microsoft and GHSL, whereas the 2019 building metrics were compared against WSF3D to maximise temporal alignment. Figure 6 illustrates building characteristics derived from the different datasets for sampled locations. We calculated Pearson’s correlation coefficient ( *r*) and the normalised root mean square difference ( *nRMSD*) to compare these datasets quantitatively. High dataset comparability was indicated by an *r* value approaching 1 and a minimised *nRMSD.* Tables 3 and 4 summarise the *r* and *nRMSD* computed in the current comparative analysis. The following discussion highlights on the results associated with *T* = 0.5 as the dataset with the best comparability with other datasets, but the scores relevant to the other thresholds were also computed.

Our dataset, especially the one produced using *T* = 0.5, exhibited the strongest agreement with the Google Polygons dataset (panel b, e, and i of Figure 6). Building count showed a high correlation ( *r* = 0.88, *nRMSD* = 0.44), while building area demonstrated the strongest correlation overall ( *r* = 0.90, *nRMSD* = 0.42). Although building perimeter derived from OBT correlated well with Google Polygons ( *r* = 0.85), a systematic deviation was observed ( *nRMSD* = 0.71). Specifically, our method tended to underestimate building perimeter compared to the Google Polygons dataset, particularly at higher values. In densely populated areas, imperfect edge detection sometimes resulted in the blending of some buildings, leading to an underestimation of total building perimeter. At the right-end of the distribution (i.e., total building perimeter of around 800 m), our method underestimated the total building perimeter of about 20% compared to Google Polygons dataset. While lowering the confidence threshold was explored as a potential solution, it did not improve accuracy. Although this adjustment reduced the distinction between neighbouring buildings, it also had the unintended consequence of further decreasing the estimated perimeter.

The high degree of agreement between the Google Polygons and Temporal datasets was anticipated, given their shared source imagery and similar (though not identical) deep learning methodologies. This concordance also suggests that the processing steps in the current study were performed effectively.

A comparison of our data products with those derived from the Microsoft dataset is summarised in Tables 3 and 4. The Microsoft dataset contains fewer buildings than OBT, which is reflected in our data showing a significantly larger total building area. This suggests that OBT may have a higher building detection rate. Notably, OBT has demonstrated high accuracy in building counts, achieving an *R*² coefficient of variation of 0.91 and a mean absolute error of 5.67 when evaluated at 300 × 300 m ^2^ tiles.

**Table 3.** Pearson’s correlation coefficient ( *r*) between 100-m resolution OBT computed using different thresholds and other datasets. Four characteristics were evaluated: building count ( *n*), perimeter ( *p*), area ( *A*), and volume ( *V*).

	Google Polygons			Microsoft			GHSL		WSF3D		
Threshold	n	A	p	n	A	p	A	V	n	A	V
0.3	0.65	0.68	0.61	0.92	0.89	0.70	0.80	0.66	0.54	0.14	0.31
0.4	0.65	0.68	0.71	0.90	0.90	0.78	0.77	0.65	0.53	0.15	0.32
0.5	0.88	0.90	0.85	0.61	0.68	0.78	0.74	0.63	0.52	0.15	0.33

**Table 4.** Normalised root mean square difference ( *nRMSD*) between 100-m resolution OBT computed using different thresholds and other datasets. Four characteristics were evaluated: building count ( *n*), perimeter ( *p*), area ( *A*), and volume ( *V*).

	Google Polygons			Microsoft			GHSL		WSF3D		
Threshold	n	A	p	n	A	p	A	V	n	A	V
*0.3*	3.11	1.56	0.64	0.54	0.76	0.81	0.83	0.78	1.01	0.79	1.42
*0.4*	2.79	1.29	0.57	0.46	0.56	0.76	0.69	0.79	0.93	0.81	1.41
*0.5*	0.44	0.42	0.71	2.33	1.04	0.50	0.60	0.81	0.84	0.84	1.41

Our data products showed a weak correlation with both the GHSL and WSF3D datasets. Figure 5, panel (f ), reveals a saturation effect in the GHSL data, with building areas at 100-m grid cells rarely exceeding 4,000 m ^2^, while OBT estimates can reach 8,000 m ^2^ or more. The correlation between OBT and WSF3D building areas was particularly weak ( *r* = 0.15). These discrepancies likely arise from differences in input imagery, computational methods, and resampling processes.

A complex relationship exists between GHSL and building volumes from the OBT ( Figure 5, panel (j)). In rural areas, where building density is low, the OBT dataset shows a larger total volume of buildings than the GHSL dataset. Conversely, the GHSL dataset records a higher total volume of buildings when considering a broader scale. Despite this, the building volumes from these two datasets maintain a moderate positive correlation ( *r* > 0.5). On the other side, the correlation between OBT and WSF3D building volumes was considerably weaker, with a normalised root mean square difference exceeding 1.

Similar to the OBT case, Sentinel-2 imagery serves as a primary input for estimating total built-up surface in both GHSL and WSF. However, the production procedures for these datasets differ. GHSL ^19^ generates built-up surfaces via symbolic machine learning, trained on an older Landsat-based GHSL dataset and combined with other sources such as the European Settlement Map, ^60^ Facebook High-Res Settlement Data, and Microsoft Building Footprints. Different sources are selected depending on the availability of the datasets in the training regions. These variations in training sets directly influence the machine learning outputs. Furthermore, textural filtering of small gaps (1–2 m) faces constraints due to the limited resolution of Sentinel-2 images, which degrades the model’s ability to infer built-up surfaces. To produce GHSL height and volume layers, a 30-m DEM is combined with the built-up surface layer to estimate average net building height. This process occurs at a 250-m resolution before resampling to a final 100-m resolution. Despite using Sentinel-2 inputs, these procedures result in smoother spatial variations in terms of building height and volume. Finally, GHSL is stored in a Mollweide projection such that reprojecting the data to the EPSG:4326 used in our study potentially introduces discrepancies.

In the case of WSF3D, a Sentinel-2-derived vegetation index identifies impervious areas ^20^ as the starting point for computing built-up fraction and area. Meanwhile, TDX-DEM data serves as the main input for building height estimation. Within identified impervious areas, height variations in the 12-m TDX-DEM indicate building edges. These values are then spatially aggregated to a 90-m grid to provide the average building height. Consequently, any inaccurate identification of the built-up area leads to errors in height estimation. Much like the GHSL case, the transformation of the WSF3D raster to our specific grid introduces additional divergence.

It is crucial to recognise that the validation process exclusively employs satellite-derived datasets, with no inclusion of authoritative ground-surveyed references. ^61^ Consequently, the presented results offer a relative validation against other datasets with similar features, and their interpretation should consider the potential for systematic biases and uncertainties inherent in remote sensing data. This mirrors the situation in other studies, such as a similar cross-comparison of building footprints covering Africa where ground-truth data was also unavailable. ^24^ Therefore, it is difficult to say which dataset is superior. Furthermore, different datasets should not be used interchangeably.

### Variance of building heights

Publicly available data concerning variance of building heights, especially for Global South, could not be found. The Global Building Morphology Indicators published by Bijecky and Chow ^31^ captured several cities, including Chennai (India), Nairobi (Kenya), and Kampala (Uganda) in the Global South. In this dataset, building indicators were computed from a building footprint dataset like the one from OSM and aggregated at administrative unit and grid-cell levels. Unfortunately, this dataset does not contain building height information for like-for-like comparison with our results.

**Figure 7.**
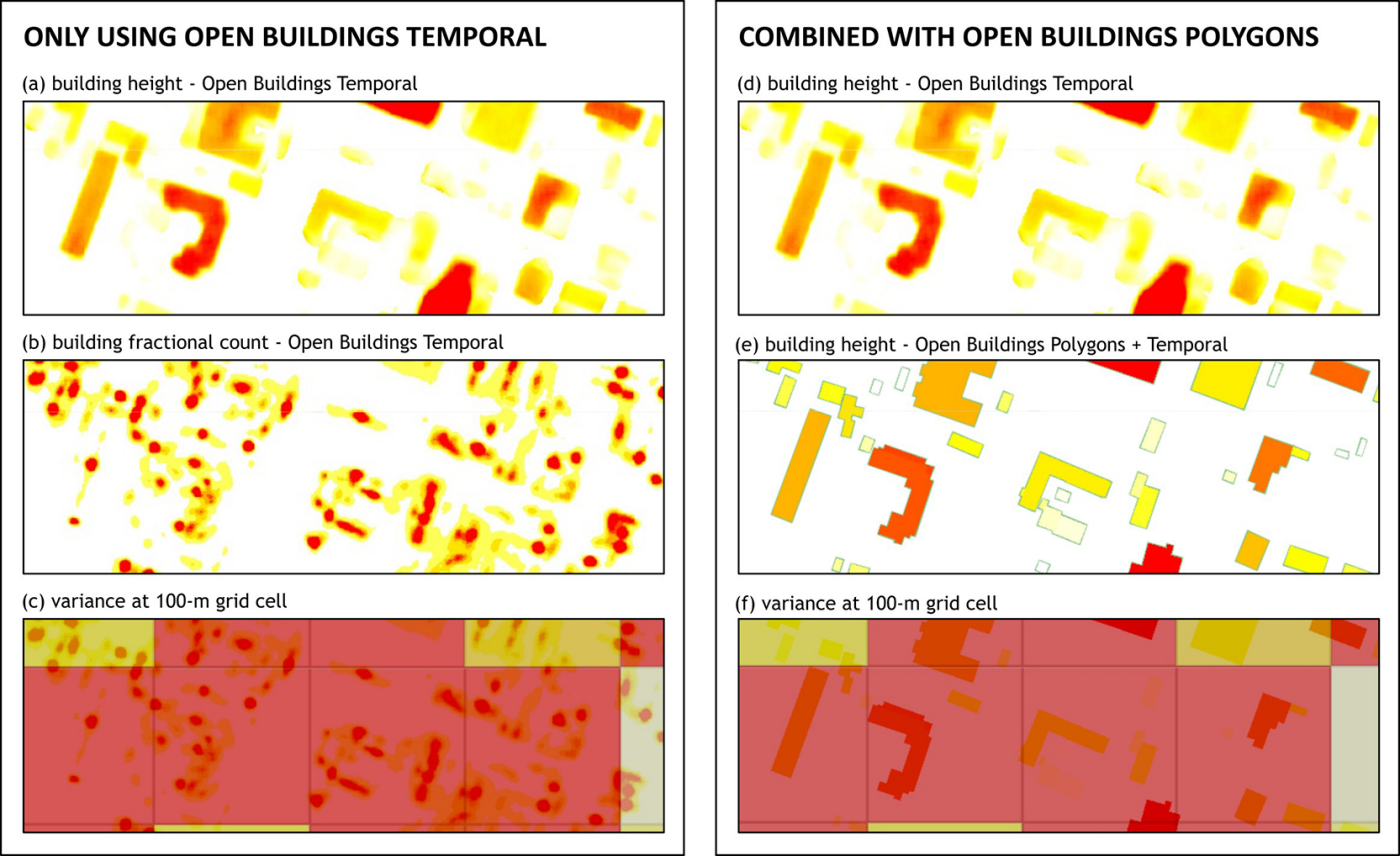
Illustration of how we computed variance of building heights using only OBT dataset (a, b, c) and in combination with Open Buildings Polygons vector dataset (d, e, f ).

Alternatively, to validate our method of computing building height variance, we combined the building height layer from OBT dataset and building footprints from Open Buildings Polygons to estimate the height for each building listed in the latter dataset. By doing this, we extended the vector dataset to include building height information so that vector-based aggregation could be performed as an alternative (see Figure 7 for illustration). For each 100-m grid cell, we selected buildings inside (or intersecting) the grid and computed the building height variance. This process was done for some sample locations in Honduras, Philippines, and Rwanda.

**Figure 8.**
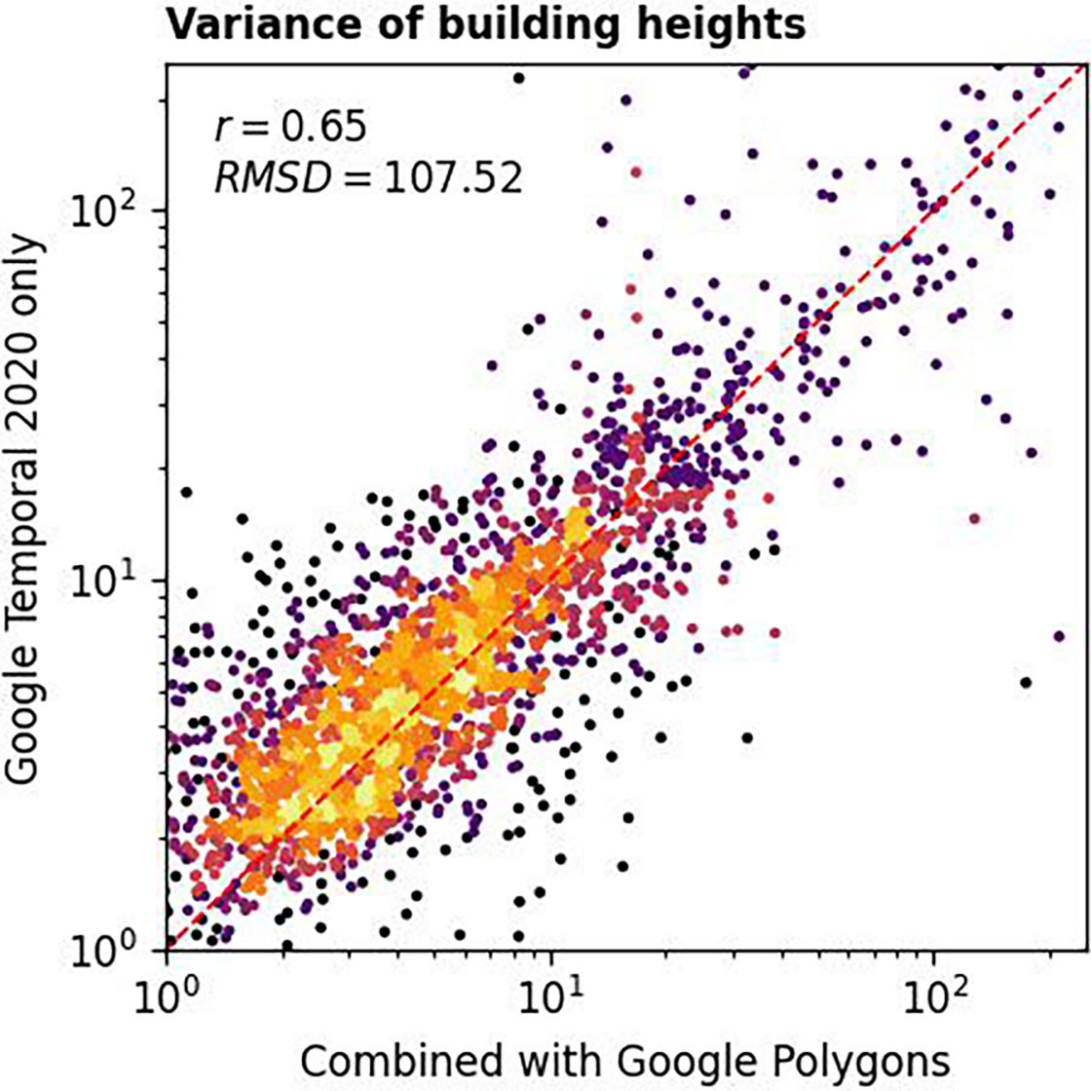
Building height variances at 183 sample locations computed using two different approaches. We used 2020 data and 0.5 threshold for this analysis. Lighter colour represents more data points.

By comparing building height variances computed using Equation 1 and the ones from the combined dataset, we demonstrated the validity of our method. Figure 8 displays how well-correlated the two were. The obtained Pearson’s correlation coefficient was 0.79 while the root mean square deviation was 45.76.

### Correlation with population count estimates

**Figure 9.**
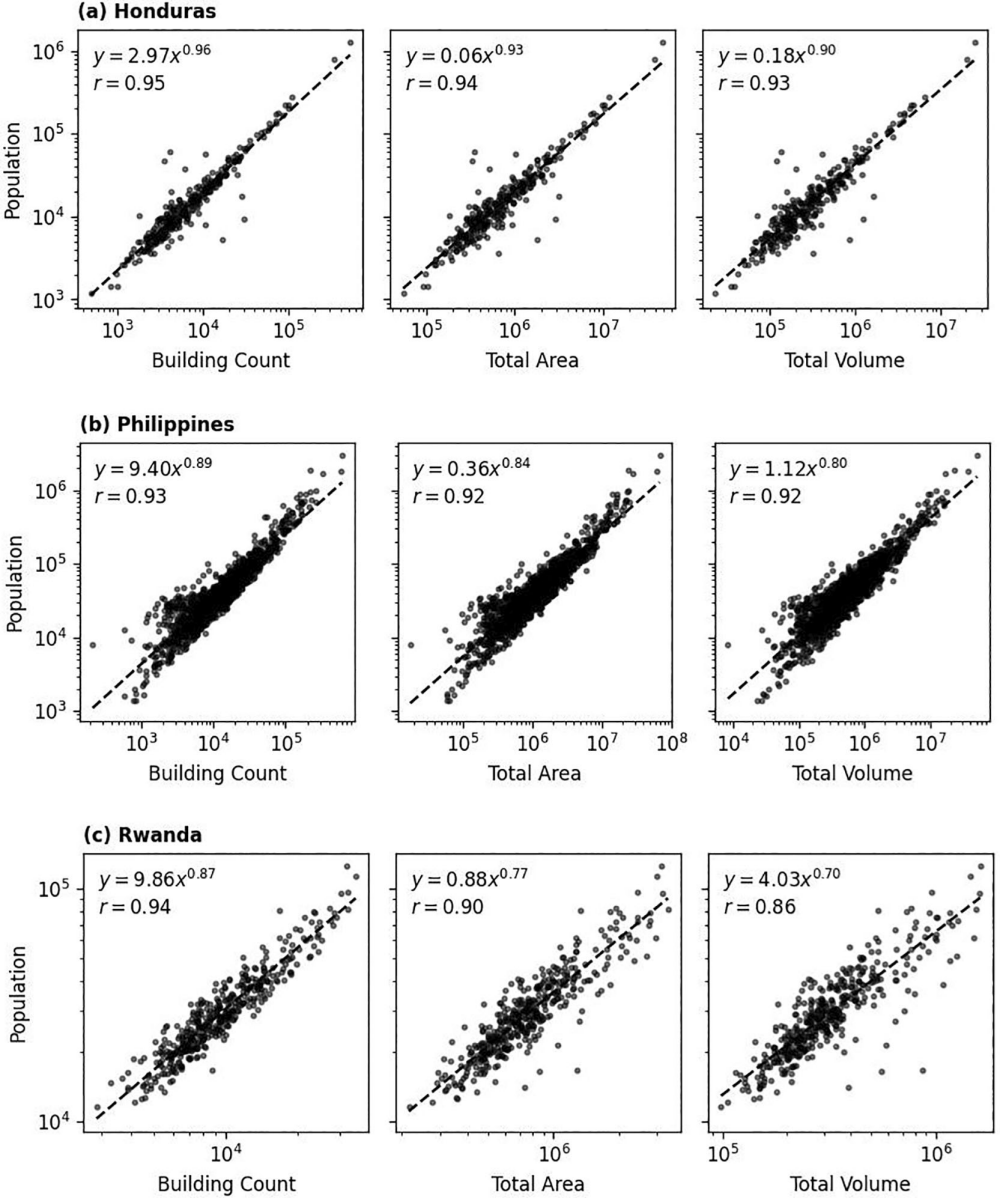
Correlation between building count, total area, and volume with total population count at certain administrative units. Exponential relation is displayed on the top-right corner of each panel, together with associated Pearson’s correlation coefficient ( *r*). Dashed line represents one-to-one relationship between variables.

In summary, a strong positive correlation ( *r* > 0.8) was observed between building count and population estimate ( Figure 9). The relationships between three building characteristics and the population estimate at administrative unit level could be represented using exponential function ( *P* = *aX ^b^*, with *X* represents building characteristic) pretty well. For most of the cases, the exponents were around 0.90, while the scaling factors differ by country.

Population count at administrative unit level was affected by many factors and building characteristics like building count was only one of the factors. But, from these simplified empirical relationships we could learn some interesting points. The exponents were less than 1, implying sub-linear relationship between the variables. This implies that while population increases as building characteristics hike, the rate of population increase slows down. This could be due to factors like population density limits, differing land use policies, or increasing non-residential buildings in denser regions.

Meanwhile, the country-specific scaling factor ( *a*) acts as a baseline population density. A country with a high value for *a* will have a greater population for the same level of building characteristics, reflecting cultural, economic, or policy factors that lead to higher population density, such as a preference for large households, smaller per-capita living space, or a greater proportion of residential vs. commercial buildings.

### Different thresholds

Thresholding was a critical step in our process and was based on the building identification confidence scores provided by the dataset. These scores were uncalibrated, ^42^ meaning that a value of 0.8 did not represent a literal 80% probability of a building's existence. Instead, the scores served primarily as a tool for relative ranking and thresholding. The reliability of these values was influenced by various environmental and technical variables, including cloud cover and imagery misalignment, and showed a systematic sensitivity to roof color. While Sirko et al. ^42^ identified 0.35 as the optimal threshold for mean Intersection over Union (mIoU) and 0.42 for total built-up area, we recommend a more conservative threshold of 0.5. This choice accounts for the lack of site-specific ground truth data and aligns with the comparative analysis presented previously.

**Figure 10.**
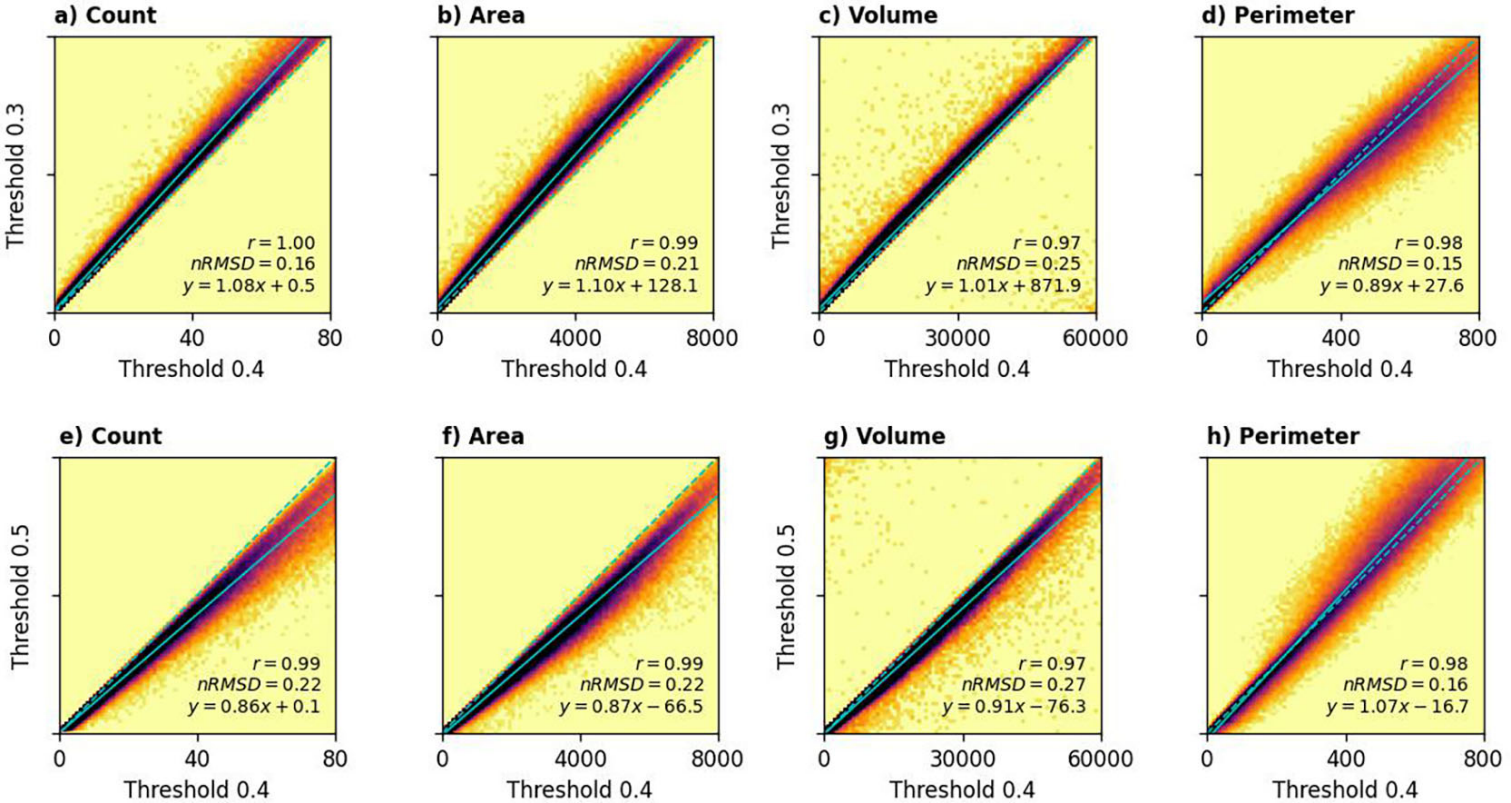
Comparison between building characteristics in sampled locations year 2020, derived using different thresholds. One-to-one relationship is represented by dashed line, while the best linear fit is marked with solid lines.

The validation described above was done using characteristics computed using T = 0.45. How do different thresholds affect the computed characteristics? Figure 10 visually summarises the answers. Higher thresholds resulted in lower building counts, areas, and volumes. This is because stricter thresholds tend to truncate building edges and exclude some fractional building counts. Conversely, a slight increase in building perimeter was observed with higher thresholds. This seemingly counterintuitive result likely stems from a balancing effect: while higher thresholds contract individual building sizes, they also reduce the blending of closely spaced buildings, thus potentially increasing the overall measured perimeter.

### Spatial consistency

In this study, we aggregated fine-resolution OBT data to a coarser 100-m resolution via spatial aggregation and additional computations. This final scale was selected to ensure compatibility with the WorldPop Global Demographic Data Project, facilitating its use in various applications at regional and global scopes. Because data aggregation can significantly influence analytical outputs and subsequent decision-making, it is critical to quantify the scale effects during spatial integration. For this purpose, we utilised GEE and extracted six building characteristics in Flor Amarillo, Carabobo State, Colombia (see Figure 3), across different spatial scales, from 5 m to 100 m. We compared pixel values at 100 m to assess the sensitivity of the result to the extraction scale and computed the Root Mean Square Difference (RMSD).

**Figure 11.**
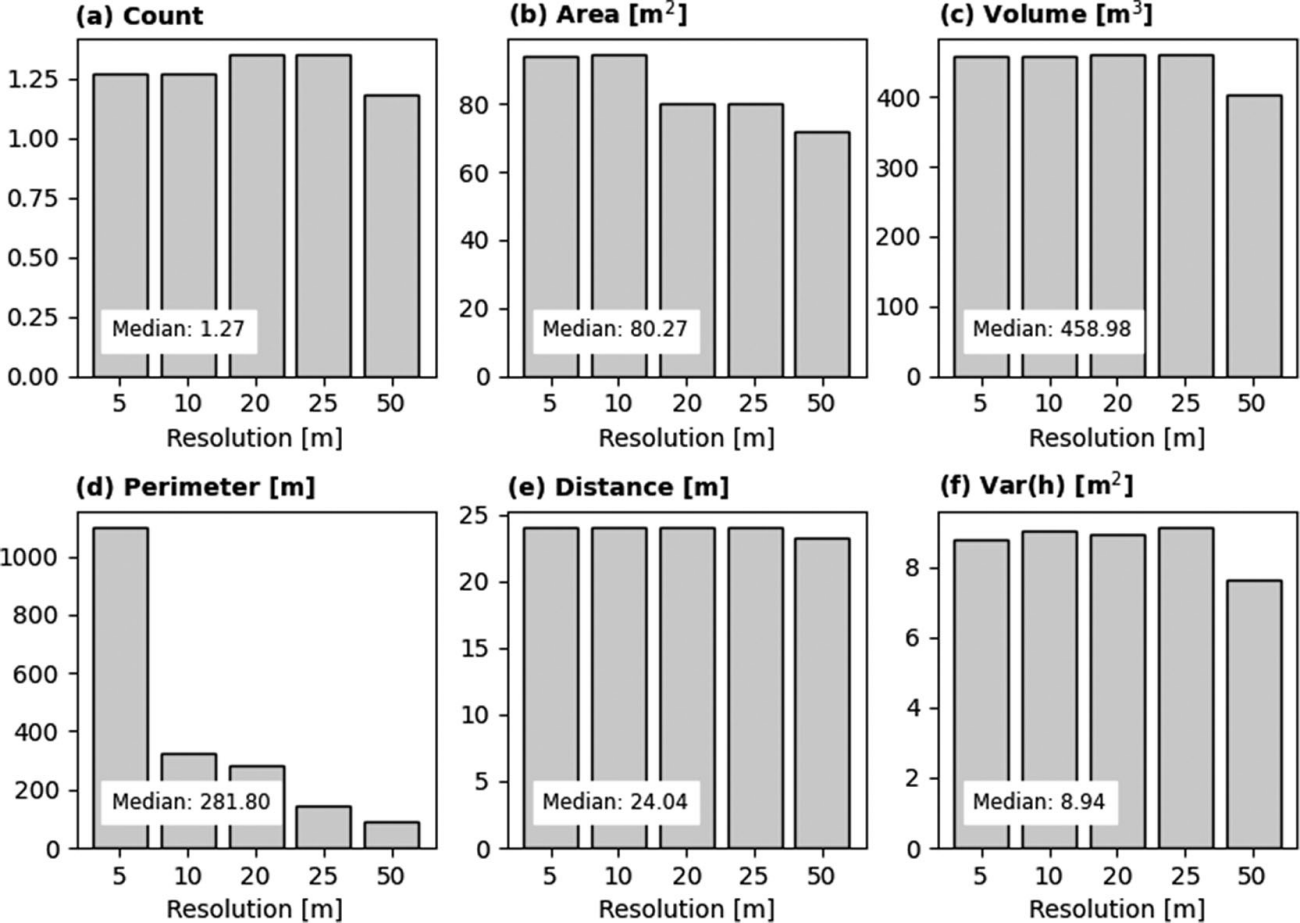
Root Mean Square Difference between building characteristics derived at 100-m and smaller resolutions.

The assessment demonstrates high compatibility between the 100 m pixel values and those derived from finer resolutions. As illustrated in Figure 11, the median *RMSD* for each building characteristic remains within acceptable thresholds, validating the consistency of the upscaling process. Notably, the median differences are relatively small, being roughly equivalent to the variance of a single building. A systematic decrease in *RMSD* is indeed observed for perimeter. Extraction at smaller scales tends to overestimate the total building perimeter due to excessive edge detection. However, this discrepancy is not a cause for concern. The 100-m total perimeter estimates produced in this study are validated against metrics derived from high-fidelity building footprint polygon data (see Figure 6).

### Temporal consistency

The temporal consistency of individual building detections presents a notable challenge. Over successive time periods, the same physical structure may be assigned varying confidence scores or height estimations. Consequently, aggregated metrics such as building counts within a defined area can also exhibit instability. Several factors contribute to this temporal variability, including: (i) the presence of cloud cover, which can obscure building features and impact detection accuracy; (ii) subtle misalignments between the input satellite imagery acquired at different times, potentially leading to inconsistencies in feature extraction; and (iii) a reduced availability of Sentinel-2 imagery, particularly during the 2016-2017 period, which limits the temporal density of observations and can affect the robustness of time-series analyses.

To evaluate temporal stability of the dataset produced, we extracted building characteristics (0.4 confidence threshold) from 183 sample locations mentioned before and examined the observed fluctuations.

We defined *x _t_* as building characteristics (e.g., building count) at time t, and its relative change as Δ = ( *x _t_*/ *x*_*t*-1_) − 1. Changes where |Δ| was below a defined tolerance were considered insignificant. To identify temporal fluctuation over an 8-year span (2016-2023), we examined the signs of the minimum and maximum Δ values calculated for each 100-m grid cell. Fluctuation was determined if sign (min(Δ)) ! = sign (max(Δ)), signifying that both positive and negative relative changes occurred, excluding any changes deemed insignificant (|Δ| < 10%).

**Figure 12.**
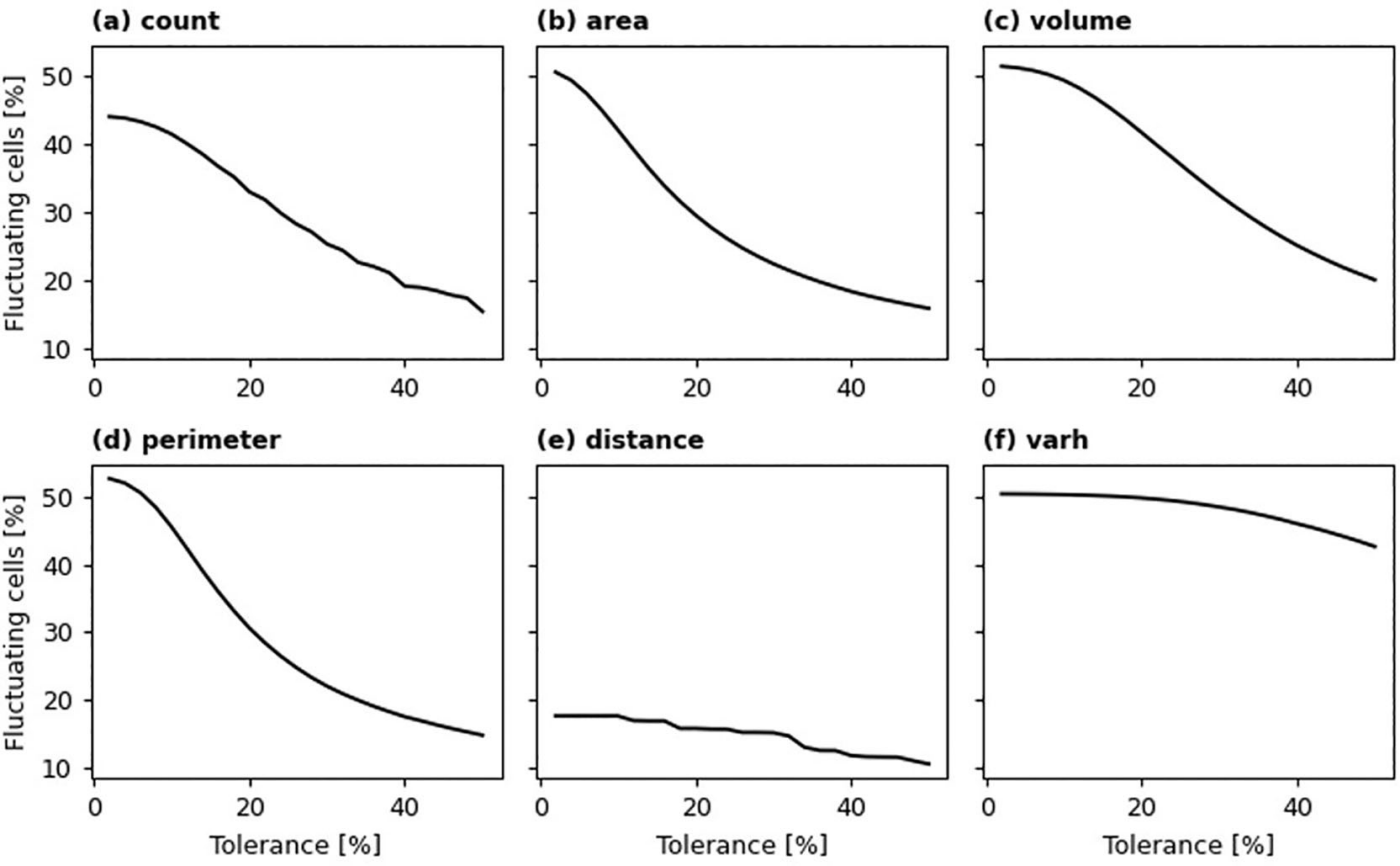
Percentage of grid cells in 183 sampled locations experiencing fluctuation at different levels of tolerance.Statistical summary of temporal fluctuation in the dataset (sampled from 183 selected locations) assuming 20% tolerance.

Following the principle outlined previously, we quantified temporal stability by calculating the percentage of grid cells exhibiting fluctuation for a given tolerance level. As illustrated in Figure 12, a lower tolerance threshold results in a higher proportion of grid cells identified as fluctuating. Specifically, for building area, volume, perimeter, and height variance ( *varh*), approximately half of the sampled grid cells show a temporal fluctuation of 5% or greater. At this same level of tolerance, around 44% of grid cells fluctuate in building count, while only about 17% display fluctuation in average building distance. The steepness of the curves in Figure 10 also indicates the temporal stability of these aggregated building characteristics. Notably, even at a higher tolerance of 20%, roughly 30% of sampled grid cells still experience fluctuation in building count, total area, and total perimeter. However, building characteristics related to height (i.e., total volume and height variance) demonstrate lower temporal stability. For height variance, the percentage of fluctuating cells remains above 40% even at a relatively high tolerance of 50%.

**Figure 13.**
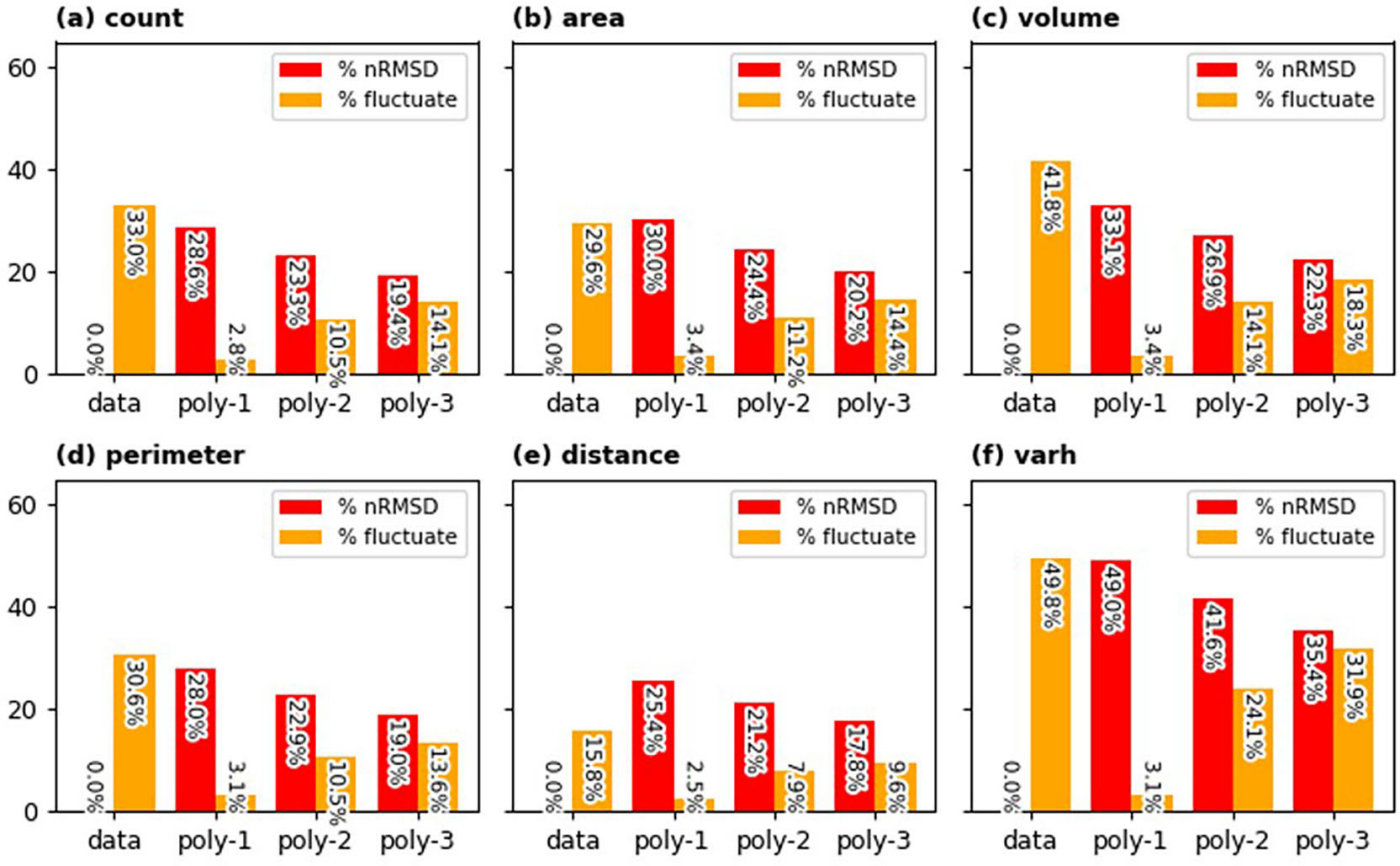
Statistical summary of temporal fluctuation in the dataset (sampled from 183 selected locations) assuming 20% tolerance. Percentages of grid cells with fluctuations in original data and the smoothed ones (using first, second, and third order polynomials) are depicted in orange bars. Emerging normalised root mean square differences ( *nRMSD*) are portrayed in red bars.

Given the inherent temporal fluctuations observed even in the aggregated dataset, further processing is necessary before utilizing this temporal data. To mitigate these inconsistencies and achieve a smoother representation of building characteristic evolution over time, we explored fitting polynomial functions to the temporal data. The outcomes of this smoothing approach are illustrated in Figure 13. In this figure, the percentage of fluctuating cells is based on 20% tolerance level while the *nRMSD* is based on the deviation between original data and the smoothed one. Fitting the first order polynomial (linear function) results in a significant reduction in fluctuating grid cells, but it introduces a large *nRMSD.* As normally expected, fitting higher order polynomials reduces the deviation between model and data, but the reduction of fluctuating cells needs to be sacrificed. Fitting second order polynomial to the data seems to be an optimal strategy to improve temporal stability while appreciating the original data.

### Uncertainty analysis

Temporal fluctuations in the dataset can also be used to quantify stochastic errors embedded in the input data set as the basis for uncertainty estimates at 100-m aggregates. For this purpose, we selected buildings from 183 sampled locations and extracted time series data representing the average building fractional count, height, and presence within a 100-meter circular buffer zone around each location, from 2016 to 2023. Values representing the building edge and building mask (where presence exceeds *T* = 0.5) were also extracted. The selection criteria required buildings to be present in 2016, as verified by both the OBT data and the World Settlement Footprint. ^20^

Assuming these locations did not undergo significant destruction or redevelopment during the study period, we expect their fractional count, height, and presence values to remain constant. Multi-temporal data can be regarded as the product of repeated measurements. Consequently, any observed temporal variations in the data are attributed to uncertainties within the OBT dataset. ^62, 63^ Standard deviation of the values (fractional count, etc.) at pixel level were computed and then spatially aggregated over the 100-m circular buffer zones to get more statistically meaningful uncertainty estimates.

**Figure 14.**
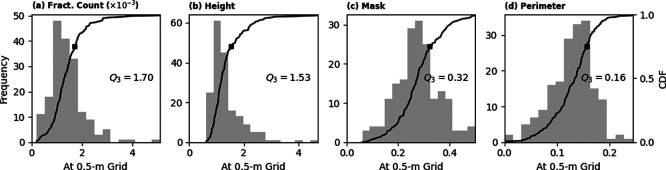
Temporal variations, parameterised as standard deviation, of building fractional count, height, mask, and perimeter extracted from 183 sample locations. Cumulative distribution functions (CDF) are indicated by black lines. Median values ( *Q*_2_) are indicated as well.

Figure 14 summarises the distributions of standard deviations representing temporal variations for the four metrics, including building mask generated through thresholding of building presence layer using *T* = 0.5. We utilise the median of these distributions as the quoted uncertainties at pixel level. The uncertainty in building height is 1.53 m, which agrees with the mean absolute error of 1.50 m reported by the OBT producer. ^42^ Estimated uncertainties for other parameters are summarised in Table 5.

**Table 5.** Uncertainty estimates of the input (pixel level) and output (100-m grid) layers.

Layer	Uncertainty	Level	Remark
Building fractional count	1.7 × 10 ^−3^	Pixel	$\sigma_{f}$
Building height	1.53 m	Pixel	$\sigma_{h}$
Building mask	0.32	Pixel	$\sigma_{m}$
Building perimeter	0.16 m	Pixel	$\sigma_{e}$
Count	1	100-m grid	$\sigma_{n}\;\approx \;200\sigma_{f}$
Total area	20 m ^2^	100-m grid	$\sigma_{A}\;\approx \;50\sigma_{m}$
Total volume	100 m ^3^	100-m grid	$\sigma_{V}\;\approx \;50\sigma_{h}$
Total perimeter	10 m	100-m grid	$\sigma_{p}\;\approx \;100\sigma_{e}$
Mean distance	-	100-m grid	
Variance of height	10 m	100-m grid	bootstrap

During the calculation process to obtain building characteristics ​​at 100-m resolution, uncertainty at the pixel level surely propagates. Assuming that the uncertainty of each variable are independent, uncertainty propagation can be calculated using the variance formula. Except for the mean distance to buildings, where the values are highly dependent to the composition and configurations of buildings, the uncertainty estimates on a 100-m grid are summarised in Table 5. A factor of $\sqrt{N}$ , with the number of original 0.5-m pixels N = 40,000 was accounted when propagating uncertainties through spatial summation and then multiplied by other factor representing the contribution of each pixel, i.e., 0.25 m ^2^ for area, 0.25 m ^3^ for volume, and 0.50 m for perimeter. Considering the complexity of the formula used to compute height variance, its uncertainty was estimated using bootstrap technique based on the uncertainties in height and fractional count. The figures summarised in Table 5 are the first order estimates of the uncertainties. The actual values may vary over diverse geographical settings.

### Usage notes

Our post-processing yielded a 100-m resolution raster of building characteristics, defined by six fundamental parameters. As indicated in literature, ^31^ aggregating building metrics or morphology at this scale offers critical insights into urban form. These metrics, including building count and total volume, facilitate a deeper understanding of both urban and rural development patterns. While the 2016–2023 OBT dataset enables the analysis of short-term residential trends, researchers must account for fluctuations inherent in multi-temporal satellite imagery. Subtle geometric misalignments between multi-temporal satellite acquisitions can introduce inconsistencies during feature extraction. Persistent cloud cover frequently results in incomplete image stacks, obscuring morphological features and degrading the accuracy of the underlying deep learning detection models. These fluctuations propagate from individual building footprints into the aggregated 100-m grid cells. Consequently, aggregated metrics can exhibit significant inter-annual fluctuations. In its raw form, the dataset requires post-processing, such as polynomial fitting or smoothing, to ensure reliability for time-series applications.

While our calculated building characteristics demonstrate high precision in stable, well-developed areas ( Table 5), significant fluctuations emerge in regions undergoing rapid growth or decline. This is particularly critical in the Global South, where small cities and rural settlements serve as hotspots for settlement expansion. ^64^ To accurately quantify regional development rates using this dataset, data fluctuations must be mitigated through robust smoothing techniques. Consequently, further investigation into optimal smoothing methodologies and their subsequent impact on results is warranted. Alternatively, computing a multi-year average prior to estimating growth would also be useful. Creation of spatio-temporal matrix ^44^ becomes another option to make use of the temporal data we produced, while maintaining monotonic growth. Though this matrix is not directly applicable to some metrics like total area and volume, spatio-temporal matrix provides information about when a particular grid cell started to be inhabited.

Tracking building heights, volumes, and 3D morphology provides deeper insights into building space inequality and urban microclimate. It is important in our endeavour to monitor progress toward the UN's Sustainable Development Goal 11 in relation to sustainable cities and communities establishment. Building volume per capita serves as a robust indicator of socio-economic well-being in certain contexts, ^26^ while spatial inequality across populations can highlight regions requiring intervention to alleviate overcrowding and improve housing. Our analysis of the relationship between population density and different building characteristics reveals that, for sampled countries in the Global South, building count serves as a more reliable proxy for population distribution than either total area or volume.

Urban microclimate modeling frequently identifies building height distribution as a critical factor. For instance, building volume density accounts for approximately 60% of the variance in local air temperatures in Beijing Metropolitan Region. ^45^ This metric can be directly derived from our data product by dividing total building volume by 100 m ^2^ grid area. Polycentricity, which defines the presence of multiple urban activity centers, is associated with a reduction in the Urban Heat Island (UHI) effect. ^65, 66^ Identification of such centers can be performed using any of the building characteristic available in our dataset. Beyond thermal impacts, variations in building height also influence the accumulation of air pollutants, specifically the concentration of particulate matter. ^45^

Lastly, remote-sensing-based building identifications often suffer from spatial uncertainties and building adhesion, which results in multiple closely-spaced buildings being represented as a single block. This pixel-to-pixel classification disregards the true morphology and regularized boundaries of individual structures, resulting in generalized, blob-like shapes. As a result, this blending inherently causes a systematic underestimation of the true building perimeter. ^67^ The building perimeter is a required mathematical input for calculating the façade-to-site ratio (FSR), another key of urban morphology parameters. Consequently, underestimating the perimeter of dense buildings leads to artificially low FSR values, miscalculation of aerodynamic roughness, and slight underestimate the severity of UHI effects. ^48^

Despite its promising potential across urban and environmental disciplines, the practical versatility of this dataset requires empirical testing in varied geographical contexts. By making this data product openly available, we encourage its application in independent studies, ensuring that it undergoes the rigorous scrutiny necessary to establish its reliability.

## Code availability

Python scripts employed to acquire and process OBT data from Google Earth Engine are available at https://github.com/rhorom/ob_25d.

## Data Availability

Under the terms of the Creative Commons License (CC BY 4.0), the final data products covering 131 countries are available at the WorldPop data repository ( DOI: 10.5258/SOTON/WP00850). More specifically, the following path leads to the file of interest: ../[TH]/[ISO]/ [band]/[iso]_buildings_[band]_[year]_glv2_5_[th]_C_100m_v1.tif Some statistical figures that summarise the building characteristics at country level are available on GitHub. All GeoTiff files are organized into directories representing country and year. Three different versions associated with three different confidence thresholds are available so that users can pick the best option for their own purposes.
